# Low *AMY1* Gene Copy Number Is Associated with Increased Body Mass Index in Prepubertal Boys

**DOI:** 10.1371/journal.pone.0154961

**Published:** 2016-05-05

**Authors:** M. Loredana Marcovecchio, Rosalba Florio, Fabio Verginelli, Laura De Lellis, Cristian Capelli, Delfina Verzilli, Francesco Chiarelli, Angelika Mohn, Alessandro Cama

**Affiliations:** 1 Department of Paediatrics, University of Chieti, Chieti, Italy; 2 Department of Pharmacy, University of Chieti, Chieti, Italy; 3 Clinical Research Centre, Centre of Excellence on Aging, University of Chieti, Chieti, Italy; 4 Department of Zoology, University of Oxford, Oxford, United Kingdom; Indiana University, UNITED STATES

## Abstract

**Background:**

Genome-wide association studies have identified more than 60 single nucleotide polymorphisms associated with Body Mass Index (BMI). Additional genetic variants, such as copy number variations (CNV), have also been investigated in relation to BMI. Recently, the highly polymorphic CNV in the salivary amylase (*AMY1*) gene, encoding an enzyme implicated in the first step of starch digestion, has been associated with obesity in adults and children. We assessed the potential association between *AMY1* copy number and a wide range of BMI in a population of Italian school-children.

**Methods:**

744 children (354 boys, 390 girls, mean age (±SD): 8.4±1.4years) underwent anthropometric assessments (height, weight) and collection of saliva samples for DNA extraction. *AMY1* copies were evaluated by quantitative PCR.

**Results:**

A significant increase of BMI z-score by decreasing *AMY1* copy number was observed in boys (β: -0.117, p = 0.033), but not in girls. Similarly, waist circumference (β: -0.155, p = 0.003, adjusted for age) was negatively influenced by *AMY1* copy number in boys. Boys with 8 or more *AMY1* copy numbers presented a significant lower BMI z-score (p = 0.04) and waist circumference (p = 0.01) when compared to boys with less than 8 copy numbers.

**Conclusions:**

In this pediatric-only, population-based study, a lower *AMY1* copy number emerged to be associated with increased BMI in boys. These data confirm previous findings from adult studies and support a potential role of a higher copy number of the salivary *AMY1* gene in protecting from excess weight gain.

## Introduction

Body mass index (BMI) is a highly heritable trait with up to 80% of its variance being attributable to genetic factors based on twin and family studies [1,2]. Over the last years a large number of common variants has been associated with BMI in genome-wide association studies, each accounting for only a small proportion of the predicted heritability [3]. This has led to the suggestion that copy number variation of genes involved in the metabolic response to diet may explain at least in part the missing heritability. In this respect the salivary and pancreatic amylase genes (*AMY1* and *AMY2*), responsible for the first-stage of starch digestion into sugar have become of increasing interest [4,5]. The copy number variation of *AMY1* seems to be evolved as an adaption to dietary habits, where larger numbers of copies have been found at higher frequencies in populations with high starch consumption in contrast to those with a low carbohydrate diet [6,7]. The first genetic link between carbohydrate metabolism and BMI has recently emerged from a large study of European and Asian adults, where the authors clearly demonstrated that reduced *AMY1* copy number was related not only to decreased salivary amylase levels, but also to increased BMI [8]. However, this association was later detected only in women with early-onset of severe obesity in a case-control study [9] and was not detected at all in a recent study performed in three large adult cohorts [10], therefore highlighting contrasting findings in this field. Results of adult studies might not necessarily be replicated in the pediatric population, especially when characterizing the genetic influence on obesity. This is supported by recent data showing a different heritability of the BMI trait during childhood and adolescence compared to adulthood [11]. However, the same study group that found an association between *AMY1* copy number and BMI in adults recently showed a protective effect of a high number of *AMY1* copies on the development of obesity also in a large case-control study of Mexican school-aged children [12]. These results suggest a potential effect of *AMY1* copy number in modulating weight gain during childhood, but this needs to be confirmed in populations from other regions, including Europe.

The aim of the present study was to assess whether *AMY1* copy number is associated with BMI z-score in a population of Italian school-children.

## Materials and Methods

### Study population

The study population was represented by Italian children attending primary schools in the town of Chieti (Abruzzo region, Central Italy).

An invitation was made to all primary schools in the local area, which all agreed to take part to the study. Out of a total school population of 779 children, 748 (96%) agreed to take part to the study.

Children were evaluated during regular school days and each school assessment was based on the same protocol, with the same examiners performing all assessments. At least one parent was asked to attend the study visit. Each visit included collection of anthropometric variables: height, weight, waist circumference and blood pressure assessment. Puberty was also assessed and staging made on the basis of breast development in girls and genital development in boys, using Tanner’s criteria.

The study was approved by the Research Ethics Committee of the University of Chieti. Written informed consent was obtained from the parents and oral assent from the children.

### Clinical assessments

Anthropometric measurements were taken according to World Health Organization recommended methods. Weight and height were measured with the child in light clothing and without shoes.

Body weight was measured to the nearest 0.1 kg with a calibrated scale (Salus, Inc., Italia).

Height was measured three times to the nearest 0.1 cm with a portable Harpenden stadiometer (Holtain, Wales, UK). Each subjects stood straight, with feet placed together and flat on the ground, heels, buttocks and scapulae against the vertical backboard, arm loose and relaxed with the palms facing medially and the head positioned in the Frankfurt plane.

A flexible tape was used to measure waist circumference to the nearest 1 mm at the mid-point between the lower ribs and the pelvic bone. Three waist circumference measurements were taken at the midst of each respiratory cycle.

### Calculations of anthropometric parameters

BMI was calculated as the weight in kilograms divided by the square of the height in meters. All anthropometric parameters were converted in z-scores using published reference values for age and sex for the Italian population [13].

### *AMY1* gene copy number

Genomic DNA was extracted from saliva by QIAmp DNA blood mini kit (Qiagen, Chatsworth, CA) as previously described [14]. *AMY1* gene copy-number was estimated by a duplex quantitative real-time PCR (qPCR) containing two TaqMan assays (Life Technologies), one for *AMY1* (Hs07226361_cn, FAM-labeled) and one specific for the reference gene (RNase P, VIC-labeled). The qPCRs were performed on Applied Biosystems 7900HT Real-Time PCR System, with Sequence Detection Software (SDS) version 2.4. As previously described [8] the assay Hs07226361_cn is specific for *AMY1* as it targets a region within exon 1 of this gene, which is absent in the amylase alpha pseudogene 1 (*AMYP1* also known as *AMY2P*). Relative copy number values were calculated by the ΔΔCT method as previously described [8] using the HapMap sample NA18956 as calibrator. This sample was included in 6 replicates for each plate and was selected as calibrator because it was consistently estimated to have 6 copies of *AMY1* by several independent methods, including whole genome-shotgun sequencing [4,7,15].

Inter-plate reproducibility for each sample was used to establish the number of replica experiments for each sample. Based on previous studies we used a cutoff of CV<17% (mean CV of 8.22%, range: 0.03–16.97) as reproducibility criterion for the inclusion of samples in statistical analyses [16,17]. Samples that did not meet this criterion in 2 replica plates were further replicated in up to 8 replica experiments and 4 samples with CV≥17% were not included in the final statistical analyses, leading to a final number of 744 subjects.

A subset of 588 samples was calibrated with both NA18956 and NA18972. NA18972 was estimated to have from 14 to 20 *AMY1* copies in different studies [4,7,15] and we considered this reference sample as having 14 copies to facilitate comparisons of our study population with those analyzed in a previous study using the same qPCR assay [8]. The overall correlation of relative copy number estimates obtained with NA18956 and NA18972 calibrators was r = 0.98 (p<0.001). For statistical analyses we used the mean of relative copy number estimates obtained using the two calibrators. Only samples (n = 506) that had CV<17% between the two estimates were included in the analyses.

### Statistical analysis

Data are expressed as means ± standard deviation (SD) or median [range].

Differences between groups were assessed with the Student's *t*-test for continuous variables, whereas categorical variables were compared with χ^2^ test or Fisher exact test. *AMY1* distributions and medians were compared between boys and girls by independent Samples Median test and Mann Whitney.

Linear regression analyses were performed to assess the association between *AMY1* copy number and BMI z-score or waist circumference. Analyses were performed using either unrounded *AMY1* relative copy number estimates, or data rounded to the nearest integer and the results were virtually identical. Data shown are those obtained using unrounded *AMY1* relative copy number estimates, unless differently specified. Analyses were adjusted for age, sex, pubertal stage, plate number, number of replications and CV%. β-coefficient was used to quantify the associations.

Statistical analyses were performed with SPSS, version 22.0 (SPSS, Inc., Chicago, Illinois). Pvalues <0.05 were taken as statistically significant.

## Results

The general characteristics of the study population are reported in Table 1. The final study population included 744 school-children (354 boys/390 girls), with a mean age (±SD) of 8.4±1.4 [range 6–11.8] years. Six hundred and forty two (86%) children were prepubertal, whereas the remaining 102 (14%) were pubertal.

**Table 1:** General characteristics of the study population.

	All	Boys	Girls	P value
**N**	744	354	390	
**Prepubertal/pubertal**	642/102	332/22	310/80	<0.001
**Age (years)**	8.4±1.4	8.5±1.4	8.3±1.4	0.08
**Height (cm)**	131.3±10.2	131.9±9.8	130.7±10.5	0.09
**Weight (Kg)**	33.7±10.6	34.1±10.4	33.5±10.7	0.43
**BMI (Kg/m**^**2**^**)**	19.2±3.8	19.2±3.8	19.2±3.9	0.97
**BMI z-score**	0.49±1.07	0.48±1.08	0.50±1.07	0.85
**Waist circumference (cm)**	62.4±9.1	63.3±9.3	61.6±8.9	0.01
***AMY1* copy number**	8.3 (1.8–27)	8.2 (2.4–21.9)	8.5 (1.8–27.2)	0.32

Data are means ±SD or median (range)

BMI = body mass index

**Fig 1:**
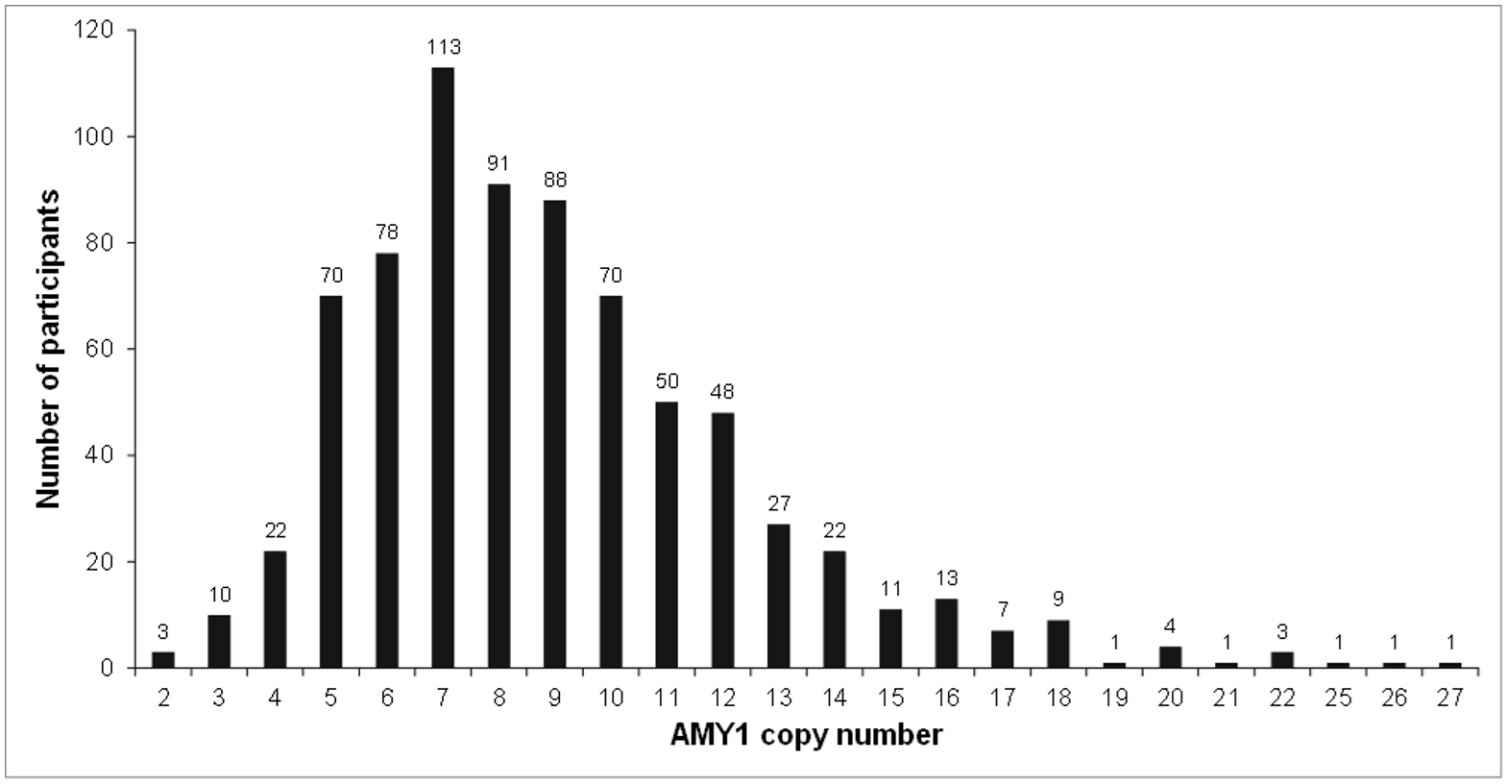
Distribution of *AMY1* copy number in the study population. Numbers on the top of columns indicate the individuals included in each copy number category. For this figure estimates of copy number have been rounded to the nearest integer.

In the whole study population the median *AMY1* relative copy number was 8.3 (range 1.8–27.2). Fourteen children (1.8%) had more than 18 copies (Fig 1).

**Fig 2:**
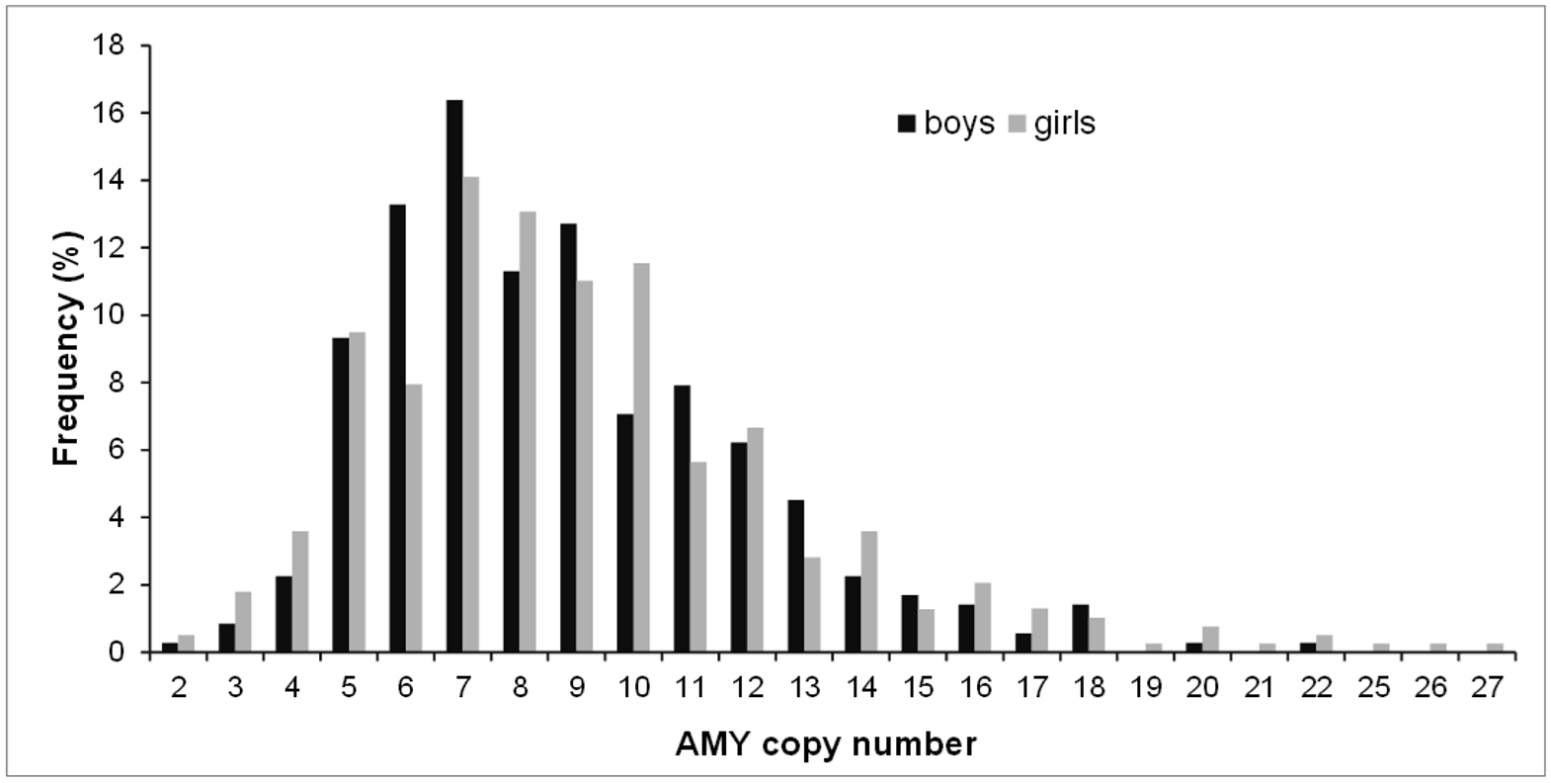
Distribution of *AMY1* copy number in boys and girls. For this figure estimates of copy number have been rounded to the nearest integer.

*AMY* relative copy number distributions and medians did not differ significantly between subgroups (Independent Samples Median test p = 0.34 and Mann Whitney test p = 0.32) (Fig 2).

**Fig 3:**
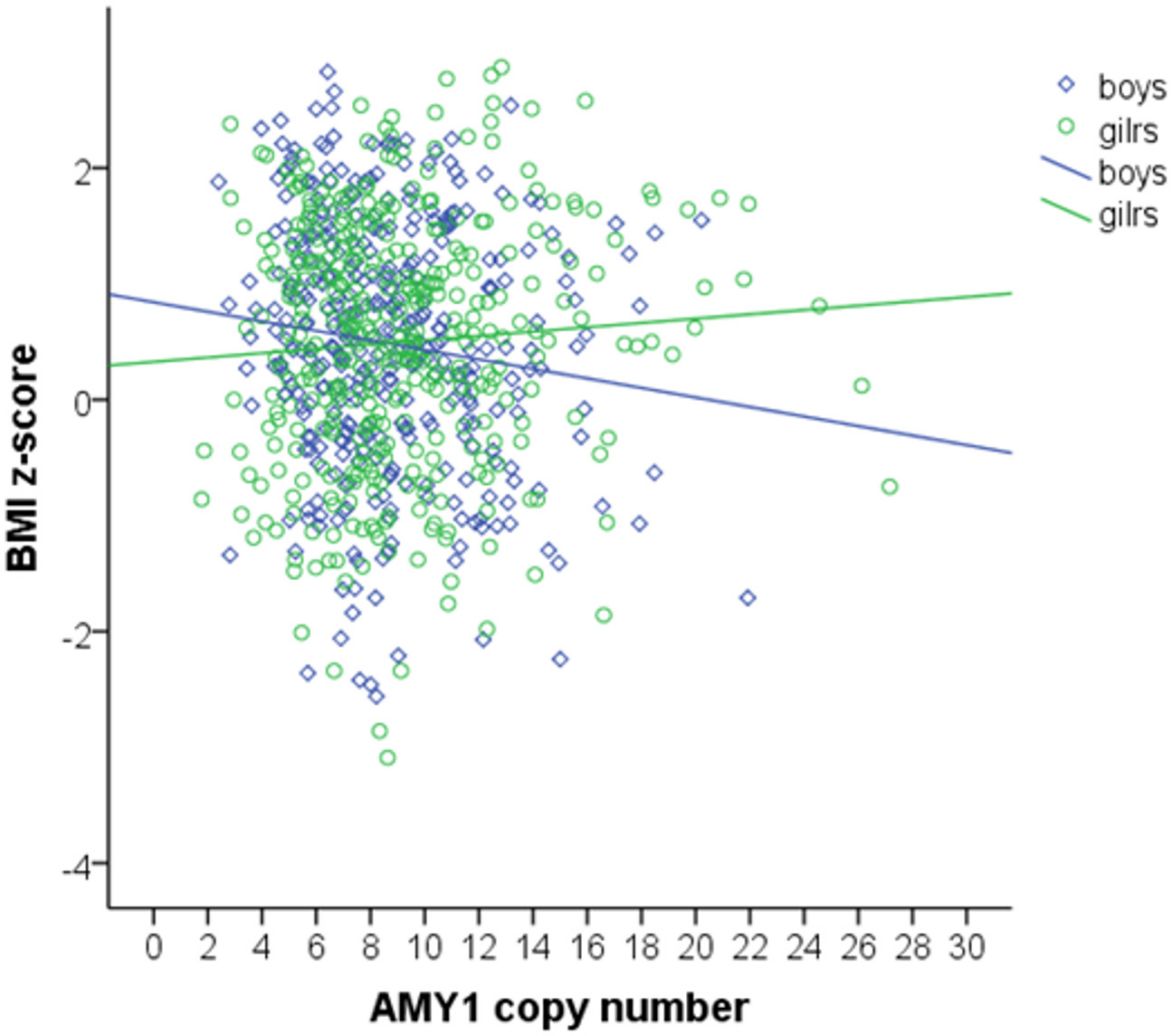
Association between *AMY1* copy number with BMI z-score in boys and girls. Linear regression analysis shows a significant reduction of BMI z-score by increasing *AMY1* relative copy number in boys, but not in girls.

No association between *AMY1* relative copy number and BMI z-score could be detected in the whole population (regression coefficient β: -0.012, p = 0.755). After dividing the study population by gender, a significant reduction in BMI z-score by increasing *AMY1* relative copy number could be found in boys (regression coefficient β: -0.117, p = 0.033), whereas the same was not detected in girls (β: 0.072, p = 0.192) (Fig 3). Similarly, waist circumference (β: -0.155, p = 0.003) was negatively influenced by *AMY1* relative copy number in boys. The results did not change after adjusting for pubertal stage.

Furthermore, boys with *AMY1* relative copy numbers below the median presented significant higher BMI z-score and waist circumference compared to boys with relative copy numbers above the median (Table 2).

**Table 2:** Anthropometric data in boys with *AMY1* copy number above or below the median.

	CNV <8	CNV≥8	P
**N**	150	204	
**Prepubertal/pubertal**	137/13	195/9	0.12
**Age (years)**	8.6±1.4	8.4±1.4	0.15
**Height (cm)**	133.1±10.5	131.1±9.1	0.06
**Weight (Kg)**	35.9±12.1	32.7±8.7	0.008
**BMI (Kg/m**^**2**^**)**	19.8±4.2	18.7±3.3	0.01
**BMI z-score**	0.62±1.07	0.38±1.08	0.04
**Waist circumference (cm)**	64.8±10.3	62.1±8.3	0.01

Data are means ±SD

BMI: body mass index

CNV: copy number variations

The association of *AMY1* relative copy number with BMI z-score and waist circumference was also assessed in the subset of 506 samples calibrated with both NA18956 and NA18972. In this subset, the significant and inverse association between *AMY1* relative copy number and both BMI z-score and circumference was confirmed in boys (β = -0.160, p = 0.014 and β = -0.185, p = 0.002, respectively), but again it was not significant in girls (β = 0.118, p = 0.08 and β = 0.074, p = 0.24, respectively). Also in this subset of the study population, the median *AMY1* relative copy number calculated using the two calibrators was 8, with a range of 2–25.

## Discussion

In this population-based study exploring the association between *AMY1* relative copy number and BMI z-score in Italian school-children we detected an independent association between low *AMY1* relative copy number and BMI z-score in boys.

One of the main strengths of the present study was the selection of a pediatric population with overall well-established living habits, especially diet. In fact, Abruzzo, a small region in the center of Italy, has a strong collective character of eating pattern, relying principally on a typical Mediterranean diet, with a clear prevalence of complex carbohydrates (almost 60%), made out of durum wheat. Furthermore, the study ascertainment was high, with less than 5% of parental refusal to participate, leading to a study population well representing the local pediatric community.

This population-based design adds precious information to the former pediatric case-control study on the association between *AMY1* relative copy number and obesity [12], as it analyses the whole spectrum of BMI variability, allowing a more reliable applicability of the results to the pediatric age-group.

*AMY1* is the gene encoding salivary amylase, the enzyme that participates in the first stage of starch metabolism [6]. Recently, a highly polymorphic copy number variation of the salivary *AMY1* gene has been investigated as a new genetic variant which could explain the missing heritability of BMI not related to single nucleotide polymorphisms [8]. However, the contribution of *AMY1* copy numbers on BMI remains controversial [10]. The first studies conducted in large adult populations indicated a clear association between *AMY1* copy numbers and increased BMI, with a significant increased risk of obesity in subjects with low compared to those with high *AMY1* copy numbers [8]. This was not confirmed in a recent study performed in three different adult cohorts [10].

Childhood data on *AMY1* copy number variations are limited, with one recent case-control study conducted in Mexican children reporting a clear global effect of *AMY1* copy number on obesity, mainly characterized by a beneficial effect of high copy numbers on reduced obesity risk [12]. A further case-control study performed in young Finnish individuals with early-onset of severe obesity confirmed a negative association between *AMY1* copy number and obesity in young women [9].

In our study population we did not detect an overall effect of *AMY1* relative copy number on BMI. However, when we performed gender-based analyses a significant reduction in BMI and waist circumference by increasing *AMY1* relative copy number variants was documented for the male gender. Boys with 8 or more *AMY1* copy number presented not only decreased BMI-z-score, but also decreased waist circumference, indicating a lower adiposity degree and potentially a better cardio-metabolic profile [18,19]. Conversely, a low copy number was associated with detrimental effects. These results are in line with the lower risk of obesity observed in the Mexican children with high copy number [12], although in that study gender-based analysis was not performed.

The results of a male-gender specific effect of *AMY1* copy number might be surprising when compared to the recently reported findings in the above-mentioned Finnish study, where *AMY1* copy number did not emerge to be a contributing factor for obesity in males, but only in females with early-onset of severe obesity [9]. However, studies with differences in populations and designs are not easily comparable, especially in the context of the intriguing genetic-environmental interplay leading to obesity. This is strongly supported by previous results from genome-wide association studies where discordant findings were detected across populations [3]. In addition, as reported for the *FTO* variant, developmental shifting might be possible especially during puberty, when hormonal changes might be the driving factors modulating the genetic background [20]. In line with this concept, prepubertal boys might exhibit a stronger genetic influence of *AMY1* copy number on BMI, which, however, might become less evident during adolescence, when relevant body composition changes occur in both genders.

In our study population the relative copy number distribution is shifted towards higher copy numbers than previously reported for other populations [8,10,12]. This might reflect in part difficulties in quantification at multi-allelic loci as highlighted by the discrepancies in copy number estimates among different laboratories in previous studies for the HapMap NA18972 Japanese sample with high copy number [4,7,10,15]. However, these difficulties are not easily overcome using methods other than the practical TaqMan assay applied in our study. In fact, even employing the less practical fibre-FISH, or whole genome sequencing read depth, NA18972 was estimated to have a variable copy number (ranging from 14 to 20), both within the same laboratory and among different laboratories [4,7,10,15]. This problem appears to affect mainly estimates of high rather than low copy numbers, since sample NA18956 used as calibrator in the present study was consistently estimated to have 6 *AMY1* copies with several different methods, including TaqMan assays, in different studies [4,7,10,15]. Although digital PCR-based methods, such as droplet digital PCR, have been proposed as more accurate methods, a small degree of uncertainty still remains for high copy number estimates also with these methods [10]. It is reassuring that *AMY1* relative copy number estimates by qPCR were highly correlated (r = 0.95; p<2.20 x 10^−16^) with those obtained by digital PCR, as elegantly demonstrated by Falchi et al. [8]. Moreover, since we used the same qPCR assay and calibrators as Falchi et al. [8], the different distribution of copy number observed in our study might also simply reflect differences among populations. This might be associated with the well-known adaptive process to starch consumption, as previously reported [6,7].

In conclusion, the present study confirms previous findings on a significant contribution of *AMY1* relative copy number on BMI also in our Mediterranean population, and extends these findings to the pediatric population. Of interest, in our pediatric population-based study the contribution of *AMY1* relative copy number was detected only in boys, suggesting a gender-specific detrimental effect of low copy number. Further studies are required to confirm this potential gender effect and its variation within generations and different populations.

## Supporting Information

S1 Table Supporting table. (PDF)
