# Supplementary material for: Low AMY1 Gene Copy Number Is Associated with Increased Body Mass Index in Prepubertal Boys
Source: PLoS One. 2016 May 5;11(5):e0154961. doi: 10.1371/journal.pone.0154961 (PMC4858278; doi:10.1371/journal.pone.0154961)
Supplement: S1 Table — (PDF) [file pone.0154961.s001.pdf]

ID	age	sex (1=male; 2=female)	BMI z-score	CN6	CV% CN6	CN14	CV% CN14	mean (CN6, CN14)	CV% (CN6, CN14)
1	7.06	1	0.3	6.92	1.96	5.85	10.18	6.39	11.90
2	7.98	1	-1.4	14.95	11.99	13.72	7.50	14.33	6.03
3	8.95	1	0.1	11.42	15.84				
4	7.60	1	-1.0	8.72	11.04				
5	8.07	2	-0.4	13.56	12.68				
6	7.44	1	2.2	5.21	6.35	4.54	1.21	4.87	9.72
7	6.13	1	-0.1	6.67	15.82	5.46	2.07	6.06	14.11
8	6.14	1	0.2	8.75	16.75	7.59	5.68	8.17	10.00
9	7.64	2	-0.3	16.78	16.62				
10	7.00	1	2.3	11.00	16.29	9.08	12.58	10.04	13.53
11	8.87	2	0.2	10.43	11.48				
12	9.65	2	0.3	11.28	14.36	10.35	9.87	10.82	6.07
13	6.32	2	1.3	11.16	1.96	9.29	15.66	10.23	12.91
14	9.07	2	0.0	7.96	1.08				
15	11.50	2	0.8	9.08	4.76	7.76	4.11	8.42	11.14
16	10.75	1	1.9	5.89	7.43	5.13	4.50	5.51	9.66
17	7.11	1	-0.5	7.55	16.01				
18	7.00	2	2.4	12.44	13.38	10.27	2.90	11.36	13.55
19	5.82	2	1.4	6.02	4.40	4.98	0.22	5.50	13.49
20	9.20	2	1.0	5.02	2.00	4.38	9.55	4.70	9.50
21	6.66	2	1.0	6.17	8.67	5.14	1.15	5.66	12.96
22	8.60	1	1.0	5.38	10.50	4.88	14.49	5.13	6.95
23	6.57	2	1.5	5.58	16.95	4.84	5.47	5.21	10.02
24	8.77	1	-0.1	12.68	16.91				
25	8.80	1	2.3	3.97	4.15	3.60	8.16	3.78	7.04
26	9.75	1	-1.1	11.85	15.33				
27	6.85	1	0.3	4.66	11.80	4.07	10.66	4.37	9.61
28	10.18	1	-0.2	11.80	10.98	10.31	11.48	11.06	9.54
29	7.53	2	0.3	8.93	10.05				
30	8.72	2	-1.4	7.70	10.09	6.70	2.54	7.20	9.82
31	6.25	2	1.6	8.18	9.44	6.59	8.72	7.39	15.20
32	6.94	2	-0.1	5.02	0.93	4.62	3.59	4.82	5.86

33	9.94	2	1.5	10.36	12.28	8.14	13.92	9.25	16.96
34	7.99	2	2.5	13.95	9.52	12.20	12.93	13.08	9.43
35	9.91	2	0.2	5.78	7.19				
36	6.45	1	0.3	10.71	2.01				
37	9.07	2	0.9	9.97	4.57				
38	8.25	2	1.2	8.62	7.49	6.80	13.35	7.71	16.75
39	7.28	2	-1.0	3.24	14.34				
40	10.60	1	-0.5	6.98	14.57	5.77	14.23	6.38	13.49
41	10.09	2	-0.3	5.32	10.81				
42	10.19	1	1.6	5.88	16.24				
43	8.04	1	-2.4	5.69	8.10				
44	7.65	1	1.0	12.37	7.15	9.77	13.01	11.07	16.60
45	6.42	2	-0.2	7.96	15.64	6.52	2.46	7.24	14.09
46	10.26	1	1.1	8.07	16.06	6.77	14.04	7.42	12.40
47	10.35	1	0.9	5.32	5.10	4.26	10.97	4.79	15.67
48	8.73	1	-0.3	8.37	4.94	7.27	8.43	7.82	9.97
49	7.86	1	1.5	10.85	15.45	8.71	13.48	9.78	15.46
50	6.90	2	-0.2	15.56	15.76	13.03	4.59	14.29	12.48
51	6.01	1	0.3	6.11	13.72	5.18	7.60	5.64	11.60
52	6.84	1	0.1	5.31	16.92	4.49	6.19	4.90	11.82
53	6.87	1	1.0	12.97	12.57				
54	9.06	2	-0.3	6.62	2.15				
55	10.42	1	1.1	4.64	15.74				
56	7.08	1	-1.1	12.11	15.22				
57	10.05	2	2.0	4.94	1.08				
58	6.52	1	-2.1	6.91	6.10				
59	9.20	2	1.5	14.12	10.52				
60	7.79	2	0.6	19.97	5.38				
61	7.66	2	-0.3	10.43	7.48	9.13	14.99	9.78	9.36
62	8.80	2	-2.0	5.46	6.67				
63	9.00	1	-0.7	7.20	11.50	6.04	0.29	6.62	12.31
64	9.59	2	2.3	8.75	13.15				
65	8.57	2	0.1	6.86	1.28	6.00	8.84	6.43	9.52

66	8.60	2	1.5	6.91	0.08	5.59	12.35	6.25	14.97
67	8.38	2	0.5	5.13	1.44	4.48	8.99	4.80	9.52
68	10.36	1	1.8	12.87	0.45				
69	7.13	2	1.8	18.30	5.39	15.27	0.59	16.79	12.77
70	10.11	1	0.5	9.45	14.52				
71	6.41	2	1.8	14.15	9.42	11.90	1.81	13.02	12.23
72	7.56	1	2.2	8.10	3.89	6.83	7.34	7.46	12.01
73	8.57	1	1.7	7.07	2.81	6.17	4.75	6.62	9.63
74	6.90	2	1.5	12.22	16.73				
75	9.06	2	1.5	5.33	15.69				
76	9.06	2	1.7	5.78	1.18	5.23	5.19	5.51	7.09
77	9.35	1	-1.3	7.39	3.02				
78	8.08	1	1.7	14.25	4.33	12.59	3.50	13.42	8.73
79	6.40	2	0.7	6.64	13.93				
80	8.28	2	2.2	7.89	13.21				
81	7.61	2	0.7	9.08	7.16	7.64	0.13	8.36	12.13
82	6.85	2	-0.7	3.53	2.93	3.25	7.44	3.39	5.80
83	9.25	1	-0.2	10.12	13.38	8.79	5.85	9.45	9.91
84	7.18	2	-2.0	12.30	10.00	11.16	7.57	11.73	6.87
85	8.25	1	-0.4	6.92	12.37	6.27	16.35	6.59	6.93
86	7.47	1	-0.7	8.72	13.96				
87	11.35	1	0.5	7.13	16.26	5.73	13.88	6.43	15.42
88	6.85	1	2.0	8.24	5.00	6.94	6.23	7.59	12.06
89	9.45	1	-2.4	7.60	5.99	7.01	10.49	7.31	5.75
90	9.26	1	1.2	15.32	1.72	13.86	5.73	14.59	7.08
91	6.18	2	0.2	5.73	2.21	4.94	7.92	5.33	10.40
92	9.54	1	-1.8	7.34	3.47				
93	7.35	2	2.6	15.93	0.71				
94	6.91	2	-0.3	7.83	2.53				
95	10.59	1	1.5	8.70	14.52				
96	8.26	1	0.2	11.24	4.53	9.37	5.29	10.31	12.82
97	9.20	1	2.2	4.75	4.57	3.96	5.26	4.36	12.82
98	5.71	2	-0.1	8.32	1.44	7.45	16.08	7.89	7.77

99	10.23	1	0.5	6.63	16.81	5.57	6.66	6.10	12.34
100	8.34	1	-0.6	6.72	9.62				
101	8.34	1	0.6	10.56	4.44	8.71	16.67	9.63	13.60
102	8.50	1	-0.4	7.24	7.29	6.31	0.27	6.78	9.75
103	9.70	2	2.9	12.83	2.94	11.61	6.95	12.22	7.06
104	7.32	2	0.1	13.94	4.29	11.81	15.47	12.88	11.69
105	8.42	2	0.4	9.33	7.18	7.88	0.38	8.60	11.87
106	11.33	2	1.1	6.54	13.07				
107	10.47	1	0.5	10.10	14.12				
108	8.06	2	1.7	8.84	14.87				
109	9.60	1	0.7	7.00	4.02	6.11	3.55	6.55	9.66
110	10.60	2	-0.7	9.36	11.52	7.78	1.72	8.57	13.06
111	6.97	2	-0.4	12.58	5.71	10.67	16.87	11.62	11.64
112	7.86	1	1.1	6.49	14.82				
113	6.48	1	0.4	6.89	7.27	5.54	7.67	6.21	15.32
114	7.20	2	0.3	10.28	11.08	8.63	0.14	9.46	12.30
115	9.99	2	1.0	6.95	7.13	6.05	0.43	6.50	9.74
116	11.12	2	1.2	5.88	16.52	4.83	10.46	5.36	13.88
117	7.50	1	0.6	4.51	5.86	3.95	13.39	4.23	9.40
118	8.00	1	-1.1	6.12	2.85	5.16	8.38	5.64	11.97
119	9.88	1	2.5	6.57	16.56	5.70	5.87	6.13	9.99
120	7.75	1	0.7	14.15	5.55	12.78	1.53	13.47	7.18
121	9.13	2	0.7	8.10	5.72	7.06	1.85	7.58	9.71
122	7.20	2	1.5	7.25	15.74				
123	7.53	2	1.6	5.77	12.59	4.66	5.90	5.21	15.08
124	7.20	2	0.3	6.04	2.96	5.38	11.45	5.71	8.15
125	7.42	1	1.3	6.80	4.00				
126	10.33	2	-0.6	7.35	10.69	6.39	3.14	6.87	9.84
127	6.65	1	-0.1	5.48	1.20	4.63	10.03	5.06	11.91
128	6.00	1	-0.9	16.56	15.11				
129	10.35	2	1.6	11.21	7.67	9.44	0.65	10.32	12.15
130	8.17	1	0.3	14.28	13.86				
131	11.31	2	-0.4	4.47	15.83				

132	8.48	2	0.1	11.73	7.33	10.63	11.33	11.18	7.00
133	7.31	2	0.5	9.55	11.87	8.34	10.59	8.95	9.62
134	8.76	2	1.9	5.02	6.17	4.39	13.70	4.70	9.39
135	8.70	2	-0.8	8.13	13.80				
136	6.53	2	0.1	9.21	4.47	7.81	15.65	8.51	11.68
137	9.09	2	0.8	11.96	7.17	10.80	3.15	11.38	7.20
138	7.38	2	1.7	8.62	14.81				
139	10.47	1	0.3	4.47	16.46				
140	7.42	2	-0.1	10.86	16.34				
141	7.54	2	-0.7	7.40	10.28	5.98	2.82	6.69	15.00
142	6.07	1	-0.2	11.57	10.83	9.83	4.69	10.70	11.54
143	6.83	1	0.6	9.32	1.37	7.87	9.86	8.60	11.91
144	10.19	2	1.0	8.65	7.41	6.82	13.27	7.74	16.71
145	7.07	2	-1.1	4.53	3.59	3.66	9.47	4.09	14.97
146	8.20	1	-1.4	7.52	0.72	6.35	10.50	6.93	11.89
147	6.75	2	1.1	8.05	2.83	6.39	1.51	7.22	16.19
148	6.92	2	1.9	5.42	2.11	4.90	1.91	5.16	7.13
149	7.23	1	0.3	6.25	2.37				
150	9.63	2	-1.0	9.82	8.72	9.05	13.21	9.43	5.70
151	7.04	2	0.1	10.42	6.38	8.78	4.85	9.60	12.11
152	6.82	1	-0.6	18.48	0.03				
153	9.20	2	1.1	11.11	6.32				
154	10.35	2	0.1	8.02	9.86	7.12	3.98	7.57	8.42
155	11.05	2	1.2	5.42	0.95	4.99	5.47	5.20	5.83
156	8.79	2	0.5	14.56	10.92	12.10	1.11	13.33	13.04
157	7.43	2	-2.3	9.12	12.66				
158	10.93	1	0.2	8.69	8.50				
159	8.50	1	-0.3	9.43	0.97	8.53	4.99	8.98	7.09
160	9.84	1	0.1	5.10	8.37				
161	6.64	2	1.1	4.43	7.94	3.73	3.29	4.08	12.17
162	10.18	2	2.2	12.49	6.27	10.40	3.55	11.44	12.88
163	9.50	2	1.4	7.06	14.04				
164	8.16	1	-1.7	8.19	2.72				

165	6.29	1	1.4	7.77	14.29	6.48	2.26	7.13	12.75
166	11.20	1	1.2	12.79	15.43				
167	7.52	2	1.1	8.67	4.88	7.97	0.36	8.32	5.92
168	8.75	1	-0.4	12.52	13.24				
169	8.27	2	1.1	10.35	9.68	9.38	13.67	9.86	6.97
170	6.45	1	1.5	8.39	8.74	7.35	13.72	7.87	9.37
171	7.20	1	1.2	6.24	11.84				
172	10.05	2	1.9	5.40	9.71	4.79	3.83	5.09	8.49
173	9.01	1	-0.2	7.24	12.30	6.56	16.28	6.90	6.93
174	9.01	1	0.9	6.80	16.63	5.45	12.61	6.13	15.55
175	8.28	1	0.0	6.93	3.17				
176	7.79	1	1.0	5.91	7.08	5.34	7.38	5.62	7.22
177	8.41	1	-1.3	8.64	9.46				
178	7.82	1	2.0	10.15	9.68				
179	7.15	1	-2.6	8.22	2.86	7.32	11.55	7.77	8.14
180	7.53	1	0.2	11.71	0.42	10.23	7.98	10.97	9.54
181	7.13	2	0.3	6.94	10.62	6.06	11.84	6.50	9.52
182	9.21	1	2.0	5.05	16.73	4.09	14.32	4.57	14.91
183	7.90	1	0.5	6.64	15.49				
184	9.17	2	0.9	7.69	2.74	6.40	7.77	7.04	12.94
185	9.66	2	-0.2	11.44	11.81				
186	6.33	1	0.8	8.91	13.93	7.76	8.52	8.33	9.78
187	9.50	2	-0.6	9.57	3.39	7.77	1.70	8.67	14.71
188	9.27	1	1.0	15.22	11.19	13.29	11.27	14.26	9.56
189	7.67	1	2.2	8.69	5.73	7.58	1.84	8.14	9.71
190	6.41	2	1.8	7.14	11.89				
191	6.70	2	1.2	6.46	11.35				
192	10.11	1	-0.5	8.23	6.19	7.23	16.24	7.73	9.16
193	9.62	2	1.7	6.39	1.95	5.34	11.75	5.87	12.59
194	8.48	2	1.3	11.35	15.66	10.73	7.80	11.04	3.94
195	7.77	2	-0.6	6.23	1.49				
196	9.50	2	-1.5	5.99	7.47	4.96	8.83	5.47	13.21
197	7.68	1	0.6	6.64	15.00				

198	6.22	2	-2.9	8.34	5.09				
199	9.09	2	-1.1	7.71	11.35				
200	9.55	2	0.2	9.66	6.65	8.42	0.91	9.04	9.73
201	10.64	2	0.5	8.11	11.53	7.49	16.00	7.80	5.66
202	10.64	1	-1.4	8.46	12.15	7.81	16.62	8.13	5.65
203	8.41	1	0.8	9.93	2.13	8.98	1.89	9.45	7.13
204	7.04	1	1.0	12.42	3.54	10.48	7.70	11.45	12.00
205	8.45	1	1.2	9.61	13.60	8.00	6.69	8.80	12.92
206	7.77	2	0.9	10.00	16.63				
207	8.58	2	1.6	7.56	6.30				
208	6.19	2	0.2	10.31	2.33				
209	7.47	2	0.5	12.87	6.96				
210	9.79	2	0.3	7.64	6.69	6.05	12.56	6.84	16.39
211	7.58	2	1.1	7.15	12.11				
212	6.60	2	-1.6	7.08	0.96	5.83	6.85	6.46	13.75
213	10.75	1	-0.4	7.76	12.73	6.71	14.88	7.24	10.24
214	6.41	1	-0.6	8.81	3.84	7.31	0.86	8.06	13.23
215	8.50	1	1.9	7.99	16.96				
216	6.55	2	0.7	5.45	10.18				
217	7.15	2	-0.9	13.91	0.60	12.80	3.92	13.35	5.85
218	6.89	2	0.0	5.85	4.87	4.69	10.74	5.27	15.56
219	10.81	1	1.3	7.98	15.07				
220	8.43	2	0.1	26.13	2.15	21.36	15.26	23.74	14.20
221	6.90	1	1.5	10.82	9.69				
222	11.24	2	0.4	9.64	16.97				
223	10.17	2	-1.0	12.30	1.05	10.75	8.61	11.53	9.53
224	8.24	1	-1.4	11.14	10.91	9.26	1.10	10.20	13.04
225	7.04	1	-1.7	21.92	3.31	17.95	3.01	19.94	14.10
226	8.14	2	-0.7	7.08	9.09				
227	7.09	2	0.3	8.19	14.64				
228	6.12	1	-0.3	9.37	9.86	7.65	9.07	8.51	14.31
229	7.27	2	-0.5	3.19	11.82	2.65	2.02	2.92	13.07
230	10.63	1	1.9	2.40	8.65	2.21	13.14	2.30	5.70

231	9.22	2	0.3	9.92	11.04	8.63	3.50	9.28	9.85
232	8.09	1	-0.3	9.26	1.72	7.54	3.75	8.40	14.51
233	9.90	1	0.3	8.73	3.50	7.62	4.06	8.17	9.65
234	6.37	1	0.4	5.42	9.66				
235	11.00	1	-1.3	2.82	2.22	2.39	13.42	2.60	11.77
236	7.30	2	2.1	4.16	13.19				
237	10.96	1	2.5	6.00	2.70	5.53	7.21	5.76	5.80
238	9.67	1	0.4	11.69	7.85	10.78	12.34	11.23	5.72
239	9.20	2	1.5	3.32	12.03	2.79	0.82	3.05	12.34
240	9.79	1	0.5	9.81	0.84	8.57	8.40	9.19	9.53
241	10.92	1	1.3	17.55	12.02	15.54	3.01	16.55	8.57
242	10.92	1	-1.3	5.23	4.78	4.44	5.51	4.83	11.63
243	11.43	2	0.7	3.98	0.16	3.54	11.12	3.76	8.25
244	8.40	2	0.5	9.18	1.20	7.66	4.69	8.42	12.72
245	8.90	2	-0.4	1.86	2.69	1.57	8.54	1.72	11.97
246	7.25	2	-1.1	16.73	16.45	14.30	16.07	15.51	11.06
247	10.17	1	-2.2	9.03	4.91	8.15	0.89	8.59	7.17
248	8.42	1	-0.4	6.13	13.45				
249	7.07	2	1.5	10.52	0.10	8.66	3.53	9.59	13.71
250	9.46	1	1.0	5.08	8.90	4.30	9.04	4.69	11.70
251	6.63	2	-0.5	16.47	13.49	13.25	13.64	14.86	15.34
252	7.30	2	-0.2	12.21	7.75	11.26	12.24	11.73	5.72
253	7.50	1	1.5	4.85	3.26	4.46	1.26	4.66	5.89
254	7.43	1	0.4	13.93	7.58	11.72	3.65	12.82	12.16
255	8.42	1	2.2	6.19	6.91	5.16	2.92	5.67	12.90
256	7.76	2	0.4	9.18	10.62	8.29	6.62	8.74	7.25
257	9.11	1	1.5	10.87	11.28				
258	7.82	2	0.6	3.42	13.63				
259	7.86	2	1.3	6.94	3.60	6.07	11.15	6.50	9.46
260	9.46	2	-0.7	5.50	6.32	4.40	3.72	4.95	15.76
261	7.80	1	-1.0	8.74	16.77	9.36	13.55	9.05	4.82
262	7.54	1	0.1	4.85	16.31	4.21	6.12	4.53	9.97
263	6.14	2	0.9	9.98	1.97				

264	8.95	2	-1.2	3.69	12.30	3.39	7.81	3.54	6.04
265	7.84	2	-0.4	8.33	3.81	7.53	0.21	7.93	7.16
266	8.92	1	-2.5	8.01	5.57	6.71	15.34	7.36	12.47
267	7.18	1	0.7	9.90	5.99	8.34	5.24	9.12	12.10
268	10.09	2	1.4	7.49	4.54				
269	7.50	1	-0.3	15.77	14.87				
270	7.60	2	0.7	11.10	8.16				
271	7.06	2	-0.7	3.95	2.78	3.64	1.73	3.79	5.89
272	6.87	1	0.2	4.89	2.87	4.13	8.36	4.51	11.97
273	10.31	1	1.7	6.05	0.34				
274	6.47	1	1.9	5.67	13.15				
275	7.30	1	0.8	9.97	9.99	9.16	5.49	9.56	6.00
276	8.24	1	1.2	5.33	16.54				
277	10.80	1	2.0	4.84	9.47	4.03	0.35	4.44	12.99
278	7.46	2	1.2	7.34	9.44	6.65	13.44	7.00	6.97
279	8.91	1	0.4	8.97	14.02				
280	9.31	2	0.6	4.99	2.38	4.20	3.51	4.59	12.15
281	8.02	1	-0.3	6.38	2.92	5.58	10.46	5.98	9.48
282	8.87	1	0.8	8.94	16.68				
283	7.68	1	-0.4	5.60	7.94	4.91	14.51	5.25	9.30
284	8.62	1	-0.6	6.06	2.45	5.29	5.11	5.68	9.62
285	7.38	2	0.5	11.53	9.96	10.03	2.41	10.78	9.82
286	9.30	1	-0.7	13.30	4.37	11.09	5.45	12.20	12.81
287	6.17	1	0.5	10.43	13.92				
288	7.80	1	0.1	13.45	3.90				
289	6.15	2	-1.1	8.04	10.88	7.03	11.58	7.53	9.54
290	7.35	1	2.0	9.24	15.54				
291	10.20	1	-0.2	7.17	6.97	6.28	14.49	6.72	9.37
292	8.18	2	1.7	7.93	0.70	7.17	4.72	7.55	7.09
293	8.35	1	1.4	14.69	1.10	12.60	10.14	13.65	10.86
294	9.74	2	1.7	20.89	12.16	18.52	9.58	19.70	8.52
295	6.44	1	0.9	5.72	12.34	5.28	16.81	5.50	5.65
296	11.40	1	0.8	7.43	10.68				

297	10.46	1	1.1	9.04	15.38	7.43	12.68	8.23	13.79
298	9.97	1	0.8	8.35	6.48	7.54	7.98	7.94	7.19
299	7.30	2	-0.2	4.27	6.18	3.59	5.05	3.93	12.10
300	10.01	1	0.0	11.05	3.91	10.18	8.42	10.62	5.78
301	9.44	1	0.0	11.69	13.61	9.47	1.13	10.58	14.85
302	8.26	2	0.4	5.79	3.35	5.24	7.36	5.51	7.06
303	8.16	1	-1.0	7.00	15.00	5.99	11.21	6.50	10.93
304	6.23	2	0.4	14.17	10.19				
305	9.88	1	1.0	7.76	3.77	6.27	9.65	7.02	15.06
306	9.30	2	1.7	14.72	2.32	13.56	6.83	14.14	5.80
307	6.00	1	2.1	10.94	16.03	10.14	9.39	10.54	5.40
308	9.21	1	-0.2	7.88	5.91	6.63	12.70	7.25	12.20
309	6.80	1	2.2	8.64	0.44				
310	8.47	2	1.5	7.92	9.42	6.89	1.87	7.41	9.81
311	10.20	2	0.4	9.42	10.74	8.70	15.21	9.06	5.67
312	7.70	1	0.0	9.31	6.92	8.59	11.41	8.95	5.73
313	10.77	2	1.0	9.39	7.87	8.48	3.86	8.94	7.21
314	7.19	2	1.3	7.52	12.11	6.81	16.09	7.16	6.93
315	6.22	2	0.9	11.19	8.43				
316	11.25	1	-0.9	7.11	4.47	6.56	8.98	6.83	5.77
317	11.02	2	0.9	5.02	16.09				
318	8.88	2	0.6	11.06	5.85				
319	7.21	1	-1.0	5.68	4.69				
320	6.47	2	0.1	6.80	9.34	5.77	3.19	6.28	11.51
321	9.34	2	2.5	10.39	11.35	8.64	1.55	9.52	13.05
322	6.60	1	0.5	12.99	5.13	10.75	0.66	11.87	13.36
323	8.93	2	1.0	7.45	7.28	6.53	14.80	6.99	9.36
324	9.23	1	2.0	11.14	11.95				
325	10.14	2	0.6	11.81	2.09	10.88	6.60	11.34	5.81
326	6.09	1	0.9	15.55	15.54				
327	10.11	1	1.5	6.54	8.38	5.73	14.08	6.13	9.34
328	8.92	1	1.9	4.60	6.71	3.83	3.11	4.21	12.89
329	6.84	1	1.5	10.75	9.14	9.70	5.13	10.23	7.23

330	8.92	2	1.0	9.45	2.37	8.54	1.65	9.00	7.14
331	11.23	2	-0.6	7.62	6.42	6.64	1.14	7.13	9.73
332	7.10	2	0.3	9.57	15.58	7.74	12.65	8.65	14.95
333	7.37	2	-0.5	10.30	6.21	9.05	16.22	9.68	9.17
334	10.92	2	-0.7	10.46	1.13	8.65	3.15	9.55	13.40
335	11.00	1	1.6	6.61	10.87	5.99	14.85	6.30	6.95
336	9.32	1	0.5	15.62	2.85	14.12	1.17	14.87	7.14
337	7.08	2	-0.1	6.61	5.91	5.97	1.89	6.29	7.19
338	8.58	1	0.7	10.71	4.06	9.68	0.04	10.19	7.16
339	6.70	1	0.3	9.65	7.16				
340	8.01	1	1.2	10.20	12.90	8.87	5.37	9.53	9.90
341	7.24	1	1.5	6.40	1.43	5.41	9.79	5.90	11.92
342	6.67	2	1.0	13.96	13.01	12.17	9.45	13.06	9.71
343	6.71	2	0.2	12.37	8.76	11.20	12.75	11.79	6.98
344	8.56	1	-0.6	10.77	9.76				
345	7.20	1	-0.4	11.78	6.96	10.26	0.60	11.02	9.74
346	6.46	1	-2.2	15.00	4.22				
347	6.67	2	1.8	7.27	15.30	6.31	7.79	6.79	9.96
348	6.90	1	1.5	17.06	5.18	14.87	2.38	15.96	9.69
349	9.88	2	0.3	12.75	5.22	11.11	2.35	11.93	9.69
350	7.46	2	-0.6	8.02	3.93				
351	7.01	1	2.8	6.42	6.22	5.35	3.61	5.89	12.88
352	7.97	2	0.1	12.08	7.80	10.58	15.31	11.33	9.35
353	6.80	1	-0.4	6.97	15.61				
354	8.98	1	1.6	11.55	13.10	10.04	5.57	10.79	9.90
355	6.76	1	0.0	11.65	15.61	9.60	10.26	10.62	13.61
356	8.30	1	1.8	4.88	2.76	4.12	3.13	4.50	11.97
357	7.31	2	-0.2	9.28	4.66				
358	7.80	1	0.4	10.10	10.81				
359	6.99	2	-1.0	5.16	16.36				
360	9.22	1	0.7	7.26	7.43	6.33	1.55	6.79	9.65
361	6.75	2	0.8	12.32	7.11				
362	8.79	2	0.5	7.52	3.92	6.27	5.90	6.90	12.80

363	9.68	1	0.4	5.14	10.02	4.66	14.02	4.90	6.96
364	8.69	1	-1.3	14.57	15.47	12.41	7.15	13.49	11.34
365	8.30	2	2.4	8.79	10.86	7.38	0.36	8.08	12.29
366	7.47	2	1.2	4.14	10.97	3.70	5.10	3.92	7.84
367	8.27	1	2.1	10.42	6.07	8.68	3.75	9.55	12.87
368	8.88	2	0.7	13.52	5.33	10.81	11.20	12.16	15.77
369	8.54	1	-0.3	7.12	1.73	6.44	5.75	6.78	7.08
370	7.78	2	1.6	19.72	2.48	17.43	1.82	18.57	8.69
371	8.42	1	-0.2	9.15	7.26	8.02	14.78	8.59	9.36
372	11.45	1	-1.2	8.77	16.55	7.30	6.21	8.03	12.86
373	6.60	2	1.3	5.14	12.86				
374	10.21	2	-0.9	14.16	1.26	11.82	8.56	12.99	12.71
375	8.53	1	1.3	6.57	15.41	5.44	13.88	6.01	13.39
376	7.60	2	1.4	5.22	6.23				
377	9.19	2	0.7	15.78	16.23	13.18	9.33	14.48	12.70
378	7.70	1	-0.9	8.18	13.37	6.63	11.78	7.40	14.84
379	7.68	1	-0.3	5.69	9.85	4.78	9.43	5.23	12.22
380	8.16	2	-0.6	7.82	11.63	7.09	15.61	7.45	6.94
381	7.30	1	2.4	4.69	1.55	4.09	9.10	4.39	9.51
382	7.60	2	0.1	6.81	7.39				
383	8.75	1	0.5	4.89	6.78	3.98	4.65	4.43	14.52
384	8.70	1	0.0	4.84	2.66				
385	9.56	1	-0.1	15.89	6.08	14.39	10.09	15.14	7.02
386	8.50	1	0.8	4.47	11.61				
387	11.30	1	0.1	7.54	1.33	6.30	11.14	6.92	12.62
388	7.60	2	-0.4	5.26	10.84				
389	8.71	2	-0.6	11.99	14.23	9.58	11.66	10.79	15.83
390	8.91	2	0.0	6.32	6.89	5.50	0.67	5.91	9.74
391	11.50	1	-0.6	13.19	15.47				
392	9.10	1	-1.1	13.14	15.72	11.41	8.21	12.28	9.97
393	10.02	2	0.9	9.64	2.42	8.07	12.22	8.86	12.58
394	10.80	2	1.3	6.61	7.62				
395	7.51	2	0.7	7.20	8.08	6.06	3.16	6.63	12.18

396	10.80	2	-1.1	4.13	16.60	3.42	6.84	3.78	13.24
397	8.29	2	2.3	11.59	5.06	10.14	12.60	10.87	9.42
398	9.58	2	0.7	7.17	1.63	6.08	9.16	6.62	11.65
399	10.40	1	0.7	9.64	6.74	8.09	16.50	8.86	12.43
400	10.56	1	1.6	9.63	1.67				
401	7.39	2	2.6	12.53	15.00				
402	10.58	1	1.9	7.78	3.34	6.46	7.96	7.12	13.15
403	6.41	2	0.4	9.76	7.42				
404	10.83	2	2.0	10.12	6.41				
405	8.25	1	1.9	6.53	1.46	5.44	4.07	5.99	12.77
406	6.87	2	-0.2	13.60	2.96				
407	6.18	1	-0.5	12.60	5.73	10.31	7.07	11.46	14.16
408	5.86	2	-0.1	4.55	15.28	3.97	9.74	4.26	9.52
409	9.09	2	-0.2	7.99	15.29	6.76	16.35	7.38	11.85
410	8.05	1	0.3	3.43	12.66	3.11	6.80	3.27	6.96
411	7.55	2	-0.9	10.60	3.38	8.58	9.26	9.59	14.87
412	8.71	2	-1.3	8.67	0.58	7.25	10.39	7.96	12.64
413	7.70	2	-1.1	5.86	2.72	4.89	7.11	5.37	12.76
414	6.80	1	2.5	13.18	14.31				
415	6.17	2	-0.8	27.16	4.08	23.36	3.51	25.26	10.63
416	10.12	1	0.3	4.67	6.22				
417	7.70	2	0.6	12.40	0.68				
418	6.50	1	0.7	5.65	5.17	4.92	2.40	5.28	9.69
419	7.97	1	1.2	9.48	13.71				
420	10.80	2	0.1	6.63	10.23	6.12	14.71	6.38	5.68
421	9.02	1	1.5	8.12	2.32	7.09	5.24	7.60	9.62
422	9.90	2	0.3	7.23	10.08				
423	6.70	1	1.0	8.75	10.35	7.35	0.88	8.05	12.27
424	7.80	2	2.2	9.19	9.77	7.72	1.45	8.45	12.25
425	8.00	2	0.9	5.22	2.04	4.42	13.24	4.82	11.78
426	6.57	1	0.5	8.08	10.93	6.79	0.29	7.44	12.29
427	9.56	1	1.4	5.46	9.56	5.04	14.05	5.25	5.69
428	8.35	2	1.8	6.21	6.33	5.22	15.03	5.72	12.33

429	6.75	1	-1.6	6.98	8.69	6.07	1.13	6.53	9.79
430	8.49	2	1.0	5.60	13.50				
431	8.45	1	0.4	7.02	6.15	6.36	10.16	6.69	7.02
432	10.50	2	2.8	12.46	10.48	10.36	0.67	11.41	13.02
433	5.74	2	-0.8	5.14	8.94	4.27	10.27	4.71	13.13
434	7.99	1	1.0	6.23	10.61				
435	5.92	2	0.6	9.13	1.73	7.53	8.62	8.33	13.56
436	6.50	2	0.6	7.63	1.84	6.89	2.18	7.26	7.13
437	6.88	2	-0.7	9.93	15.33	8.04	11.43	8.99	14.93
438	9.03	1	0.2	10.86	3.07	9.67	12.88	10.27	8.21
439	7.54	2	-1.3	12.40	3.58				
440	8.42	2	1.7	9.75	16.56				
441	6.41	2	-0.8	10.00	5.46				
442	7.67	1	1.0	8.51	0.14				
443	6.92	1	1.2	12.41	6.30				
444	9.84	2	1.1	9.33	11.96				
445	8.14	2	1.7	13.13	2.20				
446	7.00	1	0.1	6.25	5.49	5.25	8.81	5.75	12.27
447	6.58	2	1.7	10.25	16.19	8.58	10.45	9.41	12.51
448	8.09	2	-0.5	7.72	15.84				
449	9.68	2	1.7	8.41	0.35	6.98	5.54	7.70	13.13
450	6.73	2	-1.1	8.58	0.64				
451	8.75	1	1.7	9.45	11.36	8.15	0.56	8.80	10.39
452	7.08	2	-0.6	12.70	8.56	11.50	12.56	12.10	6.98
453	8.60	1	0.0	8.54	8.53	7.84	4.03	8.19	5.98
454	7.81	2	1.1	6.60	15.50				
455	10.90	1	0.9	8.24	16.12				
456	10.95	2	1.0	6.88	12.94				
457	7.52	1	1.1	9.33	6.13				
458	9.20	1	0.1	6.79	5.66	5.65	4.16	6.22	12.86
459	9.48	2	0.1	11.34	5.13	9.56	2.04	10.45	12.02
460	6.05	1	0.3	11.77	6.47	10.33	15.97	11.05	9.19
461	8.70	1	0.4	6.91	1.30	5.84	12.51	6.38	11.81

462	6.25	2	-1.6	10.97	16.54				
463	8.43	2	0.6	9.50	1.11	8.59	5.13	9.05	7.09
464	6.69	2	-0.3	6.57	11.97	5.51	0.76	6.04	12.33
465	9.14	1	1.1	8.68	11.87	7.87	15.85	8.28	6.94
466	7.58	2	0.5	5.98	8.65	5.03	15.52	5.51	12.14
467	9.97	1	0.0	5.75	1.11	5.29	3.41	5.52	5.86
468	7.09	2	0.2	12.56	11.13				
469	11.32	1	1.5	10.96	15.19				
470	6.76	2	0.6	7.97	9.58				
471	10.83	1	-0.8	12.39	8.69	10.91	4.72	11.65	8.96
472	9.08	1	0.8	8.76	7.57	6.90	13.43	7.83	16.78
473	8.30	2	1.0	9.75	12.47	8.48	4.94	9.11	9.89
474	6.67	1	1.6	8.10	2.98	7.24	11.82	7.67	7.94
475	6.90	1	0.1	6.29	8.84	5.48	1.29	5.88	9.79
476	9.12	1	0.0	7.51	6.98				
477	6.81	1	0.3	13.88	5.75	12.79	10.25	13.34	5.75
478	10.36	2	1.0	10.30	9.39				
479	7.81	2	-1.1	10.36	2.45	9.36	1.57	9.86	7.14
480	6.16	1	1.6	20.21	6.45				
481	7.49	2	0.7	11.79	4.16	10.85	0.36	11.32	5.91
482	8.93	2	1.6	16.23	15.72				
483	6.83	1	-0.1	13.43	9.35	12.17	13.35	12.80	6.97
484	10.71	1	-1.0	5.02	3.21	4.19	6.61	4.60	12.77
485	10.70	2	-2.3	6.66	5.62	5.72	14.01	6.19	10.67
486	7.80	2	-0.4	8.61	14.20	7.13	12.53	7.87	13.28
487	6.13	1	-1.3	11.30	3.77	9.45	9.38	10.38	12.61
488	9.30	1	-0.3	7.91	12.40	6.87	4.87	7.39	9.89
489	9.64	2	-0.4	9.68	0.70	8.91	5.21	9.29	5.83
490	8.03	1	0.4	9.84	1.78	8.32	12.99	9.08	11.79
491	8.42	2	1.3	14.76	10.57	13.38	14.56	14.07	6.95
492	9.20	2	1.8	7.84	16.96				
493	9.91	2	0.0	4.39	12.31				
494	9.10	2	2.4	2.83	12.62				

495	9.10	2	1.6	5.69	1.05				
496	7.17	2	-1.2	10.78	5.71	9.14	16.88	9.96	11.64
497	11.78	1	2.0	6.93	2.16	6.26	1.87	6.60	7.13
498	6.96	2	-1.1	10.27	1.21				
499	7.41	2	1.3	8.96	16.80				
500	7.42	1	1.4	5.87	0.64	4.89	5.25	5.38	12.99
501	10.75	1	-0.7	7.09	2.69	6.28	10.88	6.68	8.59
502	8.66	1	1.1	7.28	16.44				
503	7.40	1	0.2	7.17	14.42				
504	6.51	2	0.6	14.10	2.04				
505	7.92	1	-0.9	6.05	16.25				
506	8.40	1	2.2	9.32	12.46	7.47	6.40	8.40	15.55
507	7.39	1	-0.2	10.24	1.60				
508	8.00	2	0.6	7.91	4.34	7.29	8.85	7.60	5.77
509	9.33	1	1.2	7.44	15.88	6.10	14.18	6.77	14.05
510	8.54	1	0.9	5.02	5.72	4.55	9.73	4.79	7.02
511	9.79	1	-0.4	5.77	2.10	5.22	1.93	5.50	7.13
512	10.35	1	1.6	11.12	10.00	9.72	12.46	10.42	9.47
513	8.44	2	0.7	7.46	0.99	6.74	3.03	7.10	7.12
514	7.30	1	0.7	9.43	1.57	7.96	9.66	8.70	11.92
515	6.75	2	1.1	16.35	13.99	13.57	4.21	14.96	13.14
516	6.00	1	-1.1	12.67	0.04	10.52	4.68	11.60	13.16
517	7.57	1	1.9	5.72	13.15	4.98	9.31	5.35	9.72
518	9.48	2	0.4	4.51	8.99	4.16	13.48	4.33	5.70
519	7.53	2	-0.9	7.23	0.62	6.02	5.27	6.62	13.00
520	10.00	1	1.7	13.94	0.73	11.68	5.40	12.81	12.45
521	10.35	2	-0.5	8.54	13.44				
522	6.38	1	0.9	8.97	10.85	7.54	0.37	8.26	12.29
523	9.56	2	-0.7	6.51	1.99	6.00	6.50	6.25	5.81
524	8.10	1	1.0	3.54	15.29	2.99	14.93	3.26	12.00
525	8.67	2	0.5	17.37	16.21	14.42	11.93	15.89	13.12
526	8.68	2	0.6	7.18	4.08	6.28	11.62	6.73	9.45
527	9.90	1	0.5	7.37	14.74				

528	8.36	2	1.0	21.78	8.73				
529	10.25	1	-0.8	10.14	13.66				
530	7.76	2	2.4	8.57	2.38				
531	9.58	1	0.7	5.63	7.07				
532	7.27	1	0.2	7.93	14.92				
533	9.35	1	-1.1	17.92	0.16	14.66	2.90	16.29	14.16
534	9.07	2	0.5	17.85	4.78				
535	9.69	2	0.2	11.24	16.33				
536	7.62	2	1.3	11.70	9.75				
537	7.40	1	0.8	3.78	4.34	3.48	0.18	3.63	5.91
538	7.03	2	1.5	7.98	9.72	7.36	14.20	7.67	5.69
539	10.52	2	-0.6	4.60	15.28				
540	7.77	1	1.8	7.40	1.72	6.72	16.12	7.06	6.78
541	8.07	1	0.8	6.45	4.16	5.50	1.73	5.97	11.28
542	8.08	1	-1.0	5.71	15.56	4.74	5.79	5.22	13.20
543	6.21	1	0.8	6.75	3.33	5.61	7.94	6.18	13.15
544	9.16	1	0.4	8.26	1.39	7.22	8.95	7.74	9.52
545	9.06	2	0.9	12.76	1.40	10.66	8.42	11.71	12.71
546	9.42	2	1.8	5.25	1.79	4.40	7.89	4.82	12.40
547	10.28	2	0.2	5.00	4.72	4.01	10.59	4.51	15.49
548	9.96	2	-1.4	6.76	15.34	5.82	0.95	6.29	10.57
549	9.02	2	1.3	4.35	16.21				
550	8.94	2	1.7	10.15	0.06	8.48	9.87	9.31	12.66
551	9.70	1	1.3	5.10	9.08				
552	8.11	1	0.5	3.57	8.33	3.23	12.33	3.40	6.99
553	8.58	2	1.6	6.97	7.72	5.83	1.42	6.40	12.51
554	9.23	1	-0.3	11.47	9.63				
555	6.18	2	2.1	3.97	8.57	3.48	13.89	3.73	9.35
556	10.13	1	1.1	8.62	3.43	7.94	7.94	8.28	5.79
557	8.07	1	1.6	10.33	5.90	9.00	1.66	9.67	9.71
558	6.65	2	1.4	4.11	12.87	3.45	1.67	3.78	12.37
559	8.79	2	-1.9	16.61	16.00				
560	9.66	2	1.2	6.07	14.53	5.04	4.75	5.55	13.16

561	7.88	2	1.9	7.88	13.46	6.85	5.93	7.37	9.91
562	7.88	1	-0.7	10.08	7.43	8.48	3.80	9.28	12.15
563	8.24	2	-0.4	11.75	9.39				
564	7.66	1	1.6	10.26	2.58	8.97	10.13	9.61	9.49
565	8.24	1	-0.9	13.07	2.60	10.64	8.48	11.86	14.51
566	7.83	2	0.9	9.12	0.67	7.59	5.22	8.36	12.97
567	9.10	1	1.9	7.45	6.38	6.44	15.56	6.95	10.27
568	7.80	2	2.0	5.64	5.97				
569	7.66	1	0.4	5.77	8.66				
570	6.64	1	2.3	6.64	7.89				
571	9.34	1	2.2	8.89	5.61	8.05	9.62	8.47	7.02
572	9.80	1	2.0	12.22	4.09				
573	6.34	1	1.8	9.44	6.01	7.72	8.62	8.58	14.17
574	7.44	1	0.8	8.15	7.60	6.42	13.46	7.29	16.80
575	7.92	1	-0.6	8.74	5.55	7.14	16.58	7.94	14.22
576	8.53	2	1.0	5.17	11.07	4.30	1.27	4.73	13.04
577	9.00	1	-0.8	14.22	0.42				
578	10.00	1	0.6	8.74	12.72				
579	9.12	1	1.0	12.45	6.92	11.27	10.93	11.86	7.01
580	11.20	2	-0.7	12.28	10.68				
581	8.68	2	1.1	11.80	15.11	10.60	7.31	11.20	7.62
582	9.72	2	1.7	6.93	14.07				
583	6.68	2	1.5	12.09	3.14	10.15	9.32	11.12	12.33
584	9.72	2	-1.5	14.07	12.55				
585	7.13	2	1.2	6.80	3.63	5.67	6.20	6.23	12.79
586	8.10	1	1.4	7.35	3.47	6.41	4.09	6.88	9.65
587	7.09	2	1.2	15.38	1.41				
588	8.15	1	-0.3	7.83	2.27				
589	8.28	1	1.6	10.99	13.31	9.58	9.14	10.29	9.73
590	7.66	2	0.6	9.71	14.67				
591	10.17	2	0.9	10.49	10.90	9.51	14.88	10.00	6.95
592	10.45	2	-1.4	5.22	7.11	4.82	11.61	5.02	5.73
593	6.66	2	-0.9	6.66	7.29	5.24	0.49	5.95	16.87

594	7.46	2	2.5	7.64	16.17	6.15	14.50	6.89	15.36
595	10.75	2	0.4	6.96	14.41	5.60	12.84	6.28	15.35
596	7.09	1	0.3	7.77	6.63	6.54	4.60	7.16	12.12
597	7.70	2	-1.1	10.87	6.87	9.15	4.36	10.01	12.13
598	9.07	2	1.0	20.33	7.20	16.59	7.50	18.46	14.33
599	8.14	2	1.1	10.65	5.46	8.51	11.33	9.58	15.83
600	8.90	1	-1.0	12.05	15.34	9.68	10.54	10.86	15.44
601	7.19	2	0.3	9.34	8.81	8.18	13.65	8.76	9.37
602	7.80	2	0.2	5.50	14.27				
603	8.50	2	1.4	5.99	15.26	5.02	4.08	5.50	12.46
604	9.22	2	0.4	19.14	2.10				
605	6.70	2	-0.7	8.39	2.97	7.10	14.16	7.74	11.74
606	8.24	1	1.6	7.45	14.83				
607	7.63	1	1.6	10.77	3.31				
608	11.00	1	1.4	6.35	4.35	5.74	8.37	6.05	7.04
609	10.60	2	2.8	10.81	1.76	9.03	8.06	9.92	12.72
610	8.84	2	-1.2	8.29	9.94	7.21	1.81	7.75	9.86
611	5.95	1	1.4	5.73	0.04	4.87	7.74	5.30	11.57
612	8.59	2	-0.3	6.26	13.95				
613	7.13	1	0.5	10.60	1.22				
614	8.36	1	0.8	7.84	14.92	6.71	16.54	7.28	11.06
615	8.75	1	1.7	8.65	13.10				
616	9.80	1	-1.0	6.15	3.54				
617	9.82	1	-1.6	7.42	13.61	6.40	2.48	6.91	10.44
618	8.96	2	0.8	5.49	0.38	4.79	7.17	5.14	9.57
619	10.86	2	0.0	9.03	11.13	7.50	1.32	8.27	13.05
620	8.77	2	0.3	8.70	16.03	7.21	6.27	7.95	13.22
621	9.61	1	1.1	6.40	8.32	5.88	3.81	6.14	5.97
622	6.91	2	0.8	24.56	13.42	20.54	4.08	22.55	12.62
623	7.75	2	1.3	7.50	7.63				
624	8.83	1	2.0	6.39	3.82	5.59	11.36	5.99	9.45
625	10.75	1	0.7	4.90	5.83	4.23	0.06	4.56	10.46
626	9.07	2	0.1	12.40	14.20	10.38	6.93	11.39	12.53

627	8.84	2	1.7	15.52	2.53	12.99	12.33	14.25	12.57
628	6.43	1	-0.3	5.81	7.71	5.09	14.73	5.45	9.29
629	10.20	2	1.3	9.43	0.62	8.52	3.40	8.97	7.11
630	9.62	1	-2.1	12.16	6.83	10.20	16.58	11.18	12.43
631	10.60	1	0.4	5.81	8.06				
632	7.17	2	-0.2	8.19	6.35	6.89	4.88	7.54	12.11
633	8.33	1	0.2	13.22	13.95	12.10	8.10	12.66	6.27
634	11.27	2	1.1	9.66	5.65	8.91	10.15	9.28	5.75
635	10.02	1	-0.3	7.09	6.45	6.52	1.94	6.81	5.94
636	9.50	2	0.0	2.96	0.42	2.60	12.26	2.78	8.96
637	9.02	2	1.0	10.13	1.50				
638	7.48	1	1.9	11.28	6.15				
639	8.17	2	-1.1	8.17	12.42				
640	8.74	1	1.4	18.50	2.70	15.05	8.58	16.77	14.56
641	9.31	2	1.9	6.51	10.94	5.66	3.39	6.08	9.85
642	9.17	2	2.1	5.48	4.01	4.79	11.56	5.14	9.45
643	7.51	2	0.8	6.41	16.44	5.24	1.31	5.82	14.17
644	7.39	2	1.4	8.61	2.83	7.94	7.34	8.28	5.80
645	7.98	2	1.7	15.57	12.83				
646	7.07	1	1.1	9.74	1.66	8.23	9.56	8.99	11.93
647	8.13	2	-1.4	6.47	1.28	5.95	3.23	6.21	5.86
648	8.75	1	0.5	8.74	5.57	7.68	16.85	8.21	9.12
649	8.18	1	0.6	7.04	2.47	6.15	5.09	6.59	9.62
650	6.48	1	1.1	7.46	6.33	5.93	12.20	6.69	16.22
651	7.00	2	0.7	8.61	14.87	7.14	5.09	7.88	13.18
652	10.05	1	-0.7	11.54	3.32	9.66	13.11	10.60	12.55
653	10.00	2	2.1	8.64	8.42	7.12	11.90	7.88	13.67
654	6.58	1	1.5	9.52	2.64				
655	7.70	1	0.8	17.94	2.54	16.50	1.98	17.22	5.88
656	6.71	1	0.6	10.54	9.63	9.52	5.62	10.03	7.24
657	8.80	1	-1.0	11.38	4.29	9.92	3.27	10.65	9.67
658	6.10	1	-0.8	8.48	1.78	6.84	0.96	7.66	15.13
659	6.23	2	2.2	10.38	10.82	8.49	16.03	9.44	14.15

660	8.93	1	1.8	7.34	15.52	6.21	9.43	6.77	11.83
661	11.27	1	0.3	9.52	15.39				
662	9.55	2	-1.8	10.87	1.72	10.00	2.80	10.43	5.87
663	7.40	2	1.6	6.18	2.44	5.39	5.12	5.78	9.62
664	10.98	1	1.5	4.50	16.64				
665	10.60	2	0.5	9.61	2.02	8.73	14.83	9.17	6.75
666	6.90	2	0.4	8.44	2.07	7.14	13.27	7.79	11.78
667	7.10	1	2.2	6.37	13.34				
668	10.38	2	-0.2	6.61	13.88				
669	6.08	2	-1.2	6.63	16.60	5.57	5.14	6.10	12.26
670	7.85	1	0.2	7.64	1.93	6.67	5.63	7.15	9.61
671	7.96	1	0.6	8.53	5.36	7.47	12.89	8.00	9.41
672	6.43	2	-1.1	7.35	4.55				
673	8.41	2	0.5	8.57	0.04				
674	7.90	1	1.6	6.23	1.65	5.73	2.86	5.98	5.87
675	9.49	2	0.6	11.38	2.34	9.57	3.55	10.48	12.17
676	6.40	1	-0.1	5.54	10.77	4.84	11.69	5.19	9.53
677	7.37	2	0.5	18.37	6.09				
678	6.50	2	0.9	6.68	16.02	5.52	13.39	6.10	13.50
679	10.18	1	1.3	13.83	7.99	12.76	12.48	13.30	5.71
680	6.50	2	-0.7	7.52	11.10	6.56	11.36	7.04	9.55
681	6.41	2	-0.2	8.46	12.26				
682	9.86	2	0.8	7.66	6.68	6.64	3.18	7.15	10.11
683	7.76	2	-0.3	7.61	6.77	6.03	12.64	6.82	16.42
684	9.12	2	1.2	5.78	10.24	4.81	0.43	5.29	13.02
685	10.20	1	0.4	7.62	4.23	7.02	8.74	7.32	5.77
686	8.72	2	1.2	7.35	12.83	6.17	1.62	6.76	12.37
687	9.20	2	1.3	13.12	3.83	11.07	7.41	12.09	12.01
688	11.00	2	1.7	21.95	1.39	18.89	7.60	20.42	10.62
689	8.68	2	1.6	8.64	5.85				
690	6.65	2	1.2	5.31	16.19				
691	9.30	1	1.3	5.49	0.54	4.59	10.35	5.04	12.64
692	8.40	2	1.2	8.09	16.42				

693	7.09	1	2.1	5.11	16.43	4.13	13.41	4.62	14.96
694	6.09	1	0.3	8.00	10.98	6.34	13.04	7.17	16.34
695	8.25	1	0.2	10.37	15.84				
696	7.26	2	-0.5	12.12	5.79				
697	7.83	2	0.8	9.70	3.95	8.09	5.88	8.89	12.80
698	9.91	2	-0.9	6.55	10.14	6.04	14.62	6.29	5.68
699	8.88	2	0.6	7.35	8.86	6.39	1.30	6.87	9.79
700	7.22	2	0.1	10.13	16.52				
701	8.56	1	0.4	12.29	4.43	10.29	14.22	11.29	12.51
702	10.50	2	0.1	7.67	4.20	7.06	8.70	7.36	5.77
703	5.81	1	0.8	2.78	15.34	2.29	7.59	2.54	13.71
704	7.28	2	-0.9	1.77	6.56	1.60	10.57	1.69	7.01
705	9.30	1	-0.9	11.09	7.72	9.65	0.16	10.37	9.76
706	7.85	2	-3.1	8.63	1.80	7.54	9.36	8.09	9.51
707	5.69	2	0.9	15.14	8.89	12.59	4.38	13.86	13.05
708	8.90	1	0.9	5.59	9.71	4.95	3.83	5.27	8.50
709	6.75	2	1.0	10.44	16.82				
710	9.86	2	0.0	8.92	3.35	8.21	1.17	8.57	5.90
711	8.20	2	-0.2	4.61	0.19	3.85	14.51	4.23	12.67
712	6.03	2	-1.5	5.19	6.28	4.51	5.03	4.85	9.94
713	9.20	2	0.6	7.29	3.23	6.72	7.74	7.01	5.79
714	7.39	1	1.2	5.77	7.97	5.32	12.47	5.54	5.71
715	9.39	2	1.8	9.56	16.25	7.52	7.27	8.54	16.88
716	8.48	2	1.7	18.41	15.84	15.87	6.27	17.14	10.48
717	6.23	2	1.7	5.76	3.21	4.88	14.40	5.32	11.73
718	6.25	2	2.1	8.86	16.02	7.44	16.66	8.15	12.31
719	10.80	1	-1.0	6.80	14.25	5.47	13.20	6.13	15.37
720	6.83	2	2.0	13.84	3.77	12.21	9.49	13.03	8.81
721	7.68	2	-0.4	8.47	0.03	7.23	8.39	7.85	11.23
722	8.90	1	-0.1	3.61	7.21	3.15	0.35	3.38	9.75
723	9.11	2	1.7	2.84	8.61	2.36	1.21	2.60	12.96
724	11.04	1	0.1	8.29	7.97	7.26	2.08	7.78	9.38
725	8.10	1	0.5	6.55	7.86				

726	9.10	1	0.9	7.22	3.91	6.02	5.91	6.62	12.80
727	8.50	2	0.3	4.69	2.38	3.99	1.55	4.34	11.27
728	10.44	2	-1.4	9.75	14.91	7.99	3.04	8.87	14.03
729	8.59	2	0.0	13.02	0.32	11.78	4.34	12.40	7.10
730	8.59	2	0.0	7.30	2.90	6.60	6.91	6.95	7.06
731	8.96	1	-0.7	9.28	3.62	8.12	11.16	8.70	9.46
732	6.34	2	-0.7	8.14	16.94	6.50	8.37	7.32	15.81
733	8.11	1	1.4	10.64	15.33	9.59	11.35	10.11	7.32
734	7.00	2	0.8	7.26	1.24				
735	8.31	2	0.6	5.39	8.38				
736	10.89	1	2.7	6.67	4.01	6.14	0.51	6.40	5.91
737	9.56	1	0.6	15.97	13.16	13.92	9.29	14.95	9.72
738	7.18	2	0.2	10.89	16.20	9.45	6.23	10.17	9.96
739	8.78	1	0.3	11.52	3.04	9.61	6.78	10.56	12.77
740	7.27	2	1.1	5.11	7.68				
741	7.32	2	1.4	17.03	15.57	13.87	10.02	15.45	14.47
742	8.70	2	-1.0	8.66	7.02	7.58	14.54	8.12	9.37
743	7.19	2	0.1	7.99	4.64				
744	7.27	2	1.6	7.02	6.55				
